# Introduction to the special issue on high-resolution X-ray diffraction and imaging

**DOI:** 10.1107/S1600576717007257

**Published:** 2017-06-01

**Authors:** Virginie Chamard, Václav Holý

**Affiliations:** a Aix-Marseille Univ, CNRS, Centrale Marseille, Institut Fresnel, Marseille, France; b Department of Condensed Matter Physics, Charles University, Prague, Czech Republic

**Keywords:** editorial, high-resolution X-ray diffraction, imaging

## Abstract

The latest virtual special issue of *Journal of Applied Crystallography* features some highlights of the 13th Biennial Conference on High-Resolution X-ray Diffraction and Imaging (XTOP 2016), held in Brno, Czech Republic, in September 2016.

The 13th Biennial Conference on High-Resolution X-ray Diffraction and Imaging (XTOP 2016) was held in Brno, Czech Republic, in September 2016. It was organized by the Czech and Slovak Crystallographic Association in cooperation with the Masaryk University, Brno, and Charles University, Prague. The Organizing Committee was supported by an International Programme Committee including about 20 prominent scientists from several European and overseas countries, whose helpful suggestions for speakers are acknowledged. The conference was sponsored by the International Union of Crystallography and by several industrial sponsors.

In total, about 150 official delegates from different countries of three continents attended our conference. The scientific programme of the conference was divided into 12 sessions and two poster evenings. The participants presented 147 accepted contributions; of these 11 were 40 min-long invited talks, 42 were 20 min oral presentations and 98 were posters.

We followed the very good experience from the previous XTOP conferences and also organized a pre-conference school intended mainly for students and young researchers. The school consisted of six 90 min tutorial lectures covering broad fields of X-ray scattering, imaging and instrumentation. The invited talks at the conference were concentrated on recent and rapidly developing subjects with the emphasis on *in situ*, **in vivo** and *operando* studies in which advanced X-ray and synchrotron instrumentation was combined with other experimental techniques. The talks devoted to X-ray (real-space) imaging discussed especially coherent (phase-sensitive) imaging techniques; the contributions devoted to more standard scattering methods (*i.e.* imaging in reciprocal space) discussed, among others, *operando* scattering from catalytic surfaces and *in situ* investigation of growth kinetics of various types of nanostructures. A large part of the talks were focused on recent developments of synchrotron instrumentation, especially in connection with the advent of hard X-ray free-electron lasers and small-emittance synchrotron infrastructures.

We continue the tradition of previous XTOPs and herein publish a selection of original contributions from the conference in this special issue of *Journal of Applied Crystallography*. The manuscripts have been subject to standard peer review according to the usual practice of the journal. The topics of the published articles cover the broad spectrum of problems discussed during the conference, highlighting in particular four specific X-ray techniques: X-ray Bragg diffraction, small-angle scattering and reflectivity, X-ray diffraction imaging, and coherent (phase-sensitive) X-ray imaging.

(i) In the Bragg diffraction group, the articles are devoted to high-resolution diffraction from thin layers (Barchuk *et al.*, 2017 ▸; Borcha *et al.*, 2017 ▸; Lomov *et al.*, 2017 ▸), to diffraction from surface acoustic waves (Vadilonga *et al.*, 2017 ▸), to diffraction from protein crystals (Morelhão *et al.*, 2017 ▸) and to X-ray diffraction theory (Scardi *et al.*, 2017 ▸; Lobach *et al.*, 2017 ▸).

(ii) Small-angle scattering is represented by two articles, dealing with ultra-small-angle scattering from precipitates in metallic alloys (Andrews *et al.*, 2017 ▸) and X-ray reflection imaging (Jiang *et al.*, 2017 ▸).

(iii) The X-ray diffraction imaging papers present the application of X-ray topography techniques to various types of samples, such as silicon dies in integrated circuit packages (Tanner *et al.*, 2017 ▸), synthetic diamond crystals (Tranh Thi *et al.*, 2017 ▸) and subsurface layers in semiconductor epitaxial layers (Swiatek *et al.*, 2017 ▸), as well as a report on how X-ray diffraction was combined with a scanning mode to investigate ferroelectric domains in epitaxial layers (Schmidbauer *et al.*, 2017 ▸).

(iv) Coherent (phase-sensitive) X-ray imaging is presented in three articles, focused on the investigation of the near-field wavefront produced by a waveguide (Zhong *et al.*, 2017 ▸), on the issue of resolution in holographic and coherent diffraction imaging (Hagemann & Salditt, 2017 ▸), and on the coherent imaging of a single semiconductor nanorod (Davtyan *et al.*, 2017 ▸).

The virtual special issue is fully open access and is available at http://journals.iucr.org/special_issues/2017/xtop2016/.

It can be stated that, in spite of the fact that X-ray diffraction is over one hundred years old, the field of X-ray high-resolution diffraction and imaging is rapidly developing and a new generation of young and very talented scientists has appeared. Therefore, the future prospects are bright and we all look forward to the next XTOP conference, organized by Cinzia Giannini and her group, which will take place in Bari, Italy, in 2018.
